# PET Imaging of Perceptual Learning-Induced Changes in the Aged Rodent Cholinergic System

**DOI:** 10.3389/fnins.2019.01438

**Published:** 2020-01-21

**Authors:** J. Miguel Cisneros-Franco, Patrice Voss, Min Su Kang, Maryse E. Thomas, Jonathan Côté, Karen Ross, Pierrette Gaudreau, David A. Rudko, Pedro Rosa-Neto, Étienne de-Villers-Sidani

**Affiliations:** ^1^Montreal Neurological Institute, McGill University, Montreal, QC, Canada; ^2^Centre for Research on Brain, Language and Music, McGill University, Montreal, QC, Canada; ^3^Douglas Mental Health University Institute, McGill University, Montreal, QC, Canada; ^4^Research Centre for Studies in Aging, McGill University, Montreal, QC, Canada; ^5^Réseau Québécois de Recherche sur le Vieillissement, Université de Montréal, Montreal, QC, Canada; ^6^Department of Biomedical Engineering, McGill University, Montreal, QC, Canada

**Keywords:** positron emission tomography, aging, auditory cortex, operant training, somatostatin, [^18^F]FEOBV, choline acetyltransferase (ChAT), vesicular acetylcholine transporter (VAChT)

## Abstract

The cholinergic system enhances attention and gates plasticity, making it a major regulator of adult learning. With aging, however, progressive degeneration of the cholinergic system impairs both the acquisition of new skills and functional recovery following neurological injury. Although cognitive training and perceptual learning have been shown to enhance auditory cortical processing, their specific impact on the cholinergic system remains unknown. Here we used [^18^F]FEOBV, a positron emission tomography (PET) radioligand that selectively binds to the vesicular acetylcholine transporter (VAChT), as a proxy to assess whether training on a perceptual task results in increased cholinergic neurotransmission. We show for the first time that perceptual learning is associated with region-specific changes in cholinergic neurotransmission, as detected by [^18^F]FEOBV PET imaging and corroborated with immunohistochemistry.

## Introduction

The rules governing experience-dependent cortical plasticity vary during the lifespan. Important structural and functional changes that occur early in life during time-limited epochs, also known as critical periods (Knudsen, 2004), perdure well into adulthood. However, the magnitude of plastic changes as well as the type of interventions that may induce plasticity, change with age. Although plasticity is still possible in the adult brain, it occurs almost exclusively in the context of learning and requires sustained attention (Bavelier et al., 2010). Enhanced cortical plasticity during adulthood can be triggered by several mechanisms including deafferentation (Chino et al., 1992; Diamond et al., 1993; Van Brussel et al., 2011), disruption in the quality or quantity of sensory input (He et al., 2006; Zhou et al., 2011), direct manipulation of neuromodulatory systems (Rokem and Silver, 2010, 2013; Kang et al., 2014; Voss et al., 2016; Chamoun et al., 2017), and perceptual learning (de Villers-Sidani et al., 2010; Mishra et al., 2014; Voss et al., 2016).

Adult neuroplasticity is partly regulated by the cholinergic system through its main neurotransmitter, acetylcholine (ACh), which facilitates attention and learning (Hasselmo and Sarter, 2011). And like cortical plasticity, the cholinergic system is subject to significant alterations as it gets older. For example, aged cholinergic cells are vulnerable to degeneration (Perry, 1980; McGeer et al., 1984) leading to functional and structural damage of cortical projections (Gibson et al., 1981; Altavista et al., 1990) that have been linked to age-related cognitive and perceptual decline (Everitt and Robbins, 1997; Schliebs and Arendt, 2011). These deficits in neuromodulatory systems likely contribute to a state of dysregulated plasticity such that both diminished (Liguz-Lecznar et al., 2015) and enhanced plasticity (Cisneros-Franco et al., 2018) may occur with aging.

Recent investigations have shown that perceptual learning is a potent inducer of robust plastic changes in the aging brain, particularly in the auditory system (de Villers-Sidani et al., 2010; Mishra et al., 2014; Voss et al., 2016). However, whether these effects are mediated by neuromodulators such as the cholinergic system remains unclear. In other words, how plastic is the aging cholinergic system itself in response to perceptual learning? The purpose of the present study was to investigate how the aging rodent cholinergic system responds to perceptual learning in the auditory domain. A promising approach to achieve this goal was to use a novel positron emission tomogaphy radioligand that allows *in vivo* quantification of cholinergic activity throughout the brain. This radioligand, [^18^F]-fluoroethoxybenzovesamicol ([^18^F]FEOBV) is a vesamicol analog that selectively binds to the vesicular acetylcholine transporter (VAChT), a protein expressed uniquely by cholinergic neurons (Kilbourn et al., 2009). [^18^F]FEOBV has been used to study cholinergic activity in the context of aging and neurodegeneration both in humans (Parent et al., 2013a; Aghourian et al., 2017; Nejad-Davarani et al., 2019) and rodents (Parent et al., 2012; Cyr et al., 2014). In the present study, we scanned LOU rats, an inbred strain of Wistar origin described as a model of successful aging (Alliot et al., 2002; Boghossian et al., 2002; Garait et al., 2005; Dubeau et al., 2011). LOU rats have a longer and healthier lifespan than many other strains (Alliot et al., 2002) due to reduced incidence of metabolic, neoplastic, and cognitive disorders (Kollen et al., 2010). Here we show for the first time that perceptual learning is associated with region-specific changes in cholinergic neurotransmission detected by [^18^F]FEOBV PET imaging in LOU rats, and that these changes are mirrored by anatomical correlates.

## Materials and Methods

### General Procedures and Study Design

All experimental procedures used in this study were approved by the Montreal Neurological Institute Animal Care Committee and follow the guidelines of the Canadian Council on Animal Care. Aged LOU rats were housed in an environment with a 12-h light/dark cycle with unrestricted access to water. Those that underwent behavioral training were lightly food deprived. A group of rats that underwent perceptual learning training (Trained group, *n* = 5, two females, 31–37 months) was compared to a control group (Untrained group, *n* = 10; eight females, 30–40 months). Trained rats were scanned after an average of 12 weeks (range 9–18) of perceptual learning (60 min/day, 5 days/week), which was preceded by two behavioral shaping phases to ensure the rats could perform the auditory perceptual learning task (see “Auditory Training” section below). Training was stopped 1 day prior to the scan acquisition. Tissue collection for histological analysis was done 1–2 days after scan acquisition.

### Auditory Training

Behavioral training consisted of three phases, all of which took place in an acoustically transparent operant training chamber (60 × 45 × 35 cm, length × width × height) contained within a sound-attenuated chamber. During phase #1, rats were trained to make a nose poke response to obtain a food reward. During phase #2, rats were trained to make a nose poke only after presentation of an auditory stimulus. Phase #3 was the actual training program, in which rats were trained to make a nose poke only for the target stimulus (a 7 kHz pure tone) and not for a foil non-target stimulus (12 kHz pure tone). The tones were 50 ms in duration (5 ms cosine ramps) presented at 60 dB SPL, stimulus presentation was randomized, and the probability of a target stimulus presentation was 20%. The protocol for the production and presentation of stimuli, data acquisition and analysis is described in Voss et al. (2016).

### Imaging Procedures

[^18^F]FEOBV was synthesized on scanning days at the Cyclotron Facility of the McConnell Brain Imaging Centre of the Montreal Neurological Institute (Canada). [^18^F]FEOBV was synthesized using a modified method (Mzengeza et al., 2007) originally described by Mulholland et al. (1993). A *levo* enantiomerically pure precursor (ABX advanced biochemical compounds GmbH, Germany) was used, labeled with fluorine-18 using a SCINTOMICS (Lindach, Germany) hotbox module, resulting in (−)-[^18^F]FEOBV, which is the only enantiomer showing affinity for VAChT (Mulholland et al., 1998). Radiochemical purity across a total of five syntheses was 97.6 ± 1.4% (mean ± SD). Total molar activity ranged from 404 to 1331 GBq/μmol, whereas the mean total activity injected was 15.8 ± 1.4 MBq.

All scans (*n* = 15) were acquired with a CTI Concorde rodent R_4_ microPET system (Siemens Medical Solutions). Rodents under general anesthesia (4% isoflurane during induction, followed by 1–2% isoflurane through a nose cone) were placed on a pre-warmed stereotaxic head holder and positioned in the center of the scanner’s field of view. Vital signs (temperature, heart rate, and blood pressure) were monitored throughout the procedure (BIOPAC physiological monitoring). Each PET session consisted of a brief 5 min transmission followed by a 60 min emission scan. The transmission scan was obtained using a rotating [^57^Co] point source. Emission scans were initiated immediately after the transmission scan with a bolus injection of 0.2 mL of [^18^F]FEOBV radiotracer in the tail vein of the rat. Transmission and emission scans were obtained using list mode acquisition. After the procedure, animals were placed under a heating light and closely monitored until full recovery.

T_2_-weighted structural MR images were also obtained *in vivo* for one rat using a 7T Bruker Pharmascan 70/16 US dedicated pre-clinical MRI system (Bruker, Billerica, MA, United States). The Pharmascan is equipped with an Avance II radiofrequency (RF) amplifier architecture. T_2_-weighed axial images were specifically acquired using a Turbo-Rapid Acquisition with Refocused Echoes (Turbo-RARE) sequence with 26 slices of 1 mm thickness and an in-plane resolution of 150 μm. Other MRI scan parameters were: TE = 30 ms, TR = 3 s, and a RARE factor of eight. A 640 mm quadrature volume transceiver RF coil was used for rat brain imaging.

### Imaging Processing and Analysis

MicroPET images were histogrammed into 27 sequential time frames of increasing duration (8 × 30 s, 6 × 1 min, 5 × 2 min, 8 × 5 min) over 60 min. Images were reconstructed using a maximum *a posteriori* (MAP) algorithm, normalized and corrected for scatter, dead time and decay. The MINC software toolbox was used to perform all image analyses^1^. Time-averaged tissue-radioactivity images were manually co-registered to the structural MR image using a seven degrees of freedom registration matrix (rigid body transformation plus one scaling constant) (Rubins et al., 2003). The microPET image outcome measure was non-displaceable binding potential (BP_ND_). BP_ND_ was calculated using a simplified reference-tissue method (SRTM) for reversible ligands at the voxel level (Gunn et al., 1997), The cerebellar cortex served as a reference region due to its negligible amounts of cholinergic markers (Schäfer et al., 1994). The resulting images were convolved using a Gaussian kernel (FWHM = 1.2 mm).

### Immunohistochemistry, Microscopy, and Data Analysis

Tissue collection and processing were performed as previously described (Voss et al., 2016). Specific antibodies used to label brain tissue were: (1) rat anti-SOM (Millipore Sigma #MAB354, 1 : 500), (2) goat anti-ChAT (Millipore Sigma #AB144P, 1 : 200), (3) donkey anti-goat [conjugated to Alexa Fluor (AF647), 1 : 800, Jackson ImmunoResearch, West Grove, PA, United States], and (4) donkey anti-rat (AF488, 1 : 800, Jackson).

A Zeiss LSM 510 Meta confocal microscope equipped with filter for green Cy2/AF488, red CY3, and infrared CY5/AF647 was used to assess fluorescence in the immunostained sections. To locate the primary auditory cortex (A1) we used the stereotaxic coordinates (Paxinos and Watson, 2013): interaural between 5.76 and 2.16 mm and Bregma between −3.24 and −6.84 mm. To quantify the positive cells, 21 digital images of A1 cortical sections were taken with a 40× objective (Zeiss LSM 510) at random locations within each A1 of both hemispheres for each animal. All quantifications were assessed in 400–500 μm wide A1 sectors (the approximate width of A1 on coronal sections) per hemisphere extending from layer 1 to the underlying white matter. Confocal images were thresholded and adjusted for brightness to maximize the dynamic range of each channel using ImageJ^2^ and Adobe Photoshop CS5 (Adobe, San Jose, CA, United States).

We determined the number of immunolabeled cells in each section of A1 using the optical dissector method (Stereo Investigator software, MBF Bioscience, Williston, VT, United States) to avoid biased sampling. These counts were then pooled and adjusted to reflect what would have been counted in the whole 40× field. Data were then recorded as an averaged value per high power field (hpf) for each animal and group. All cells displaying labeling above background levels were counted, regardless of their staining intensity. Data from both hemispheres were pooled. An observer blind to the group membership of the animals performed all cell counts.

### Data and Statistical Analysis

Regional brain differences in [^18^F]FEOBV BP_ND_ were assessed using a regions of interest (ROI)-based analysis, via a repeated measures model with group (trained, *n* = 5; untrained, *n* = 10) as the predictor variable, and ROI and hemisphere (left or right) as within-subject variables. The effects of group and ROI were calculated with a repeated-measures ANOVA. Overall and layer-based group differences in ChAT- and SST-positive cell counts were assessed in a subset of rats (trained, *n* = 3; untrained, *n* = 6) using a two-way repeated-measures ANOVA with group and layer as factors. Group × ROI/layer multiple comparisons were corrected with Tukey–Kramer’s test.

## Results

**FIGURE 1:**
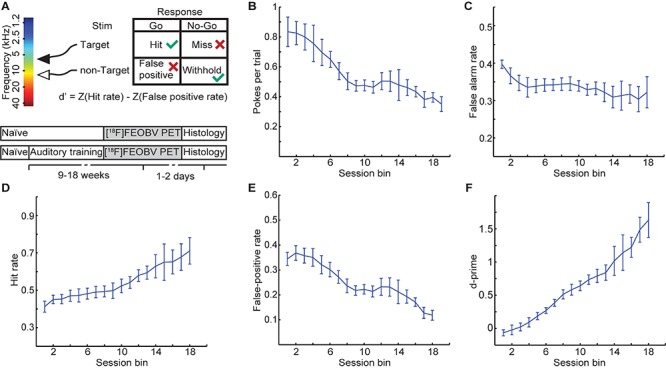
Behavioral training results on an auditory discrimination training task. **(A)** Experimental paradigm. Both **(B)** poke rate and **(C)** false alarm rate decreased with training, suggesting that rats successfully learned the association between auditory stimuli and food reward. Group behavioral performance, as measured by **(D)** hit rate, **(E)** false-positive rate, and **(F)** d-prime. Values are mean ± SEM, *n* = 5 rats.

Through training, rats learned to perform a nose poke only for the target stimulus (7 kHz pure tone) and not for a foil non-target stimulus (12 kHz pure tone; Figure 1A). The behavioral results illustrated in Figures 1B,C show that both poke rate (the number of pokes per trial) and false-alarm rate (spontaneous pokes not preceded by any auditory stimuli) decreased over time, suggesting adequate procedural learning; i.e., rats successfully learned the association between training stimuli and rewards. All trained rats showed learning effects and generally improved steadily over the training period (Figures 1D–F). Prior to undergoing a PET scan, all trained animals had reached a criterion of d-prime >1, suggesting adequate perceptual learning; i.e., successful discrimination between target and non-target stimuli.

**FIGURE 2:**
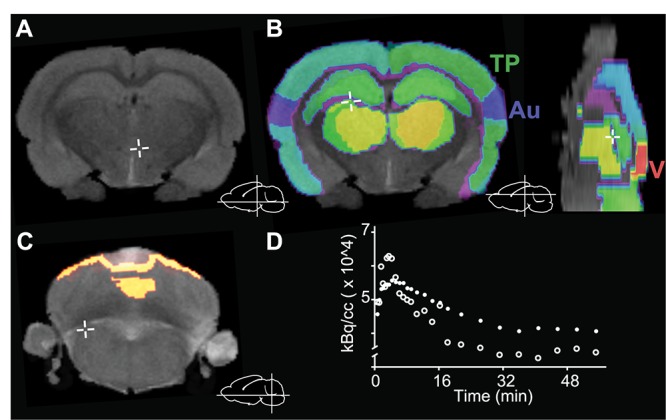
Image processing and anatomical structure segmentation. **(A)** 7T *in vivo* MR images were obtained and **(B)** used for manual segmentation into nine regions of interest (ROIs). **(C)** The cerebellum was used as reference region for data analysis. **(D)** Representative time activity curve of the reference region (cerebellum, open circles) and the rest of the brain (all ROIs except cerebellum, filled circles). Au, auditory cortex; TP, temporoparietal cortex; V, visual cortex. Insets **(A–C)** depict the position of the cursor (cross) for each panel.

In order to quantify [^18^F]FEOBV distribution in the rat brain, the following ROI were segmented using high resolution MR images of (see Figure 2): (1) frontal cortex; (2) temporoparietal cortex; (3) ventro-orbital cortex; (4) visual cortex; (5) auditory cortex; (6) nucleus basalis (7) striatum; (8) hippocampus, and (9) thalamus. The regional non-displaceable binding potential (BP_ND_) in all rats/scans was computed for each ROI in each hemisphere (resulting in 18 ROIs per rat/scan). An ROI of the cerebellum was also traced manually and used as the reference region for BP_ND_, because of its negligible amounts of cholinergic markers (Schäfer et al., 1994). In agreement with previous reports, the regions with the highest tracer binding were the striatum and nucleus basalis (Parent et al., 2012; Cyr et al., 2014).

**FIGURE 3:**
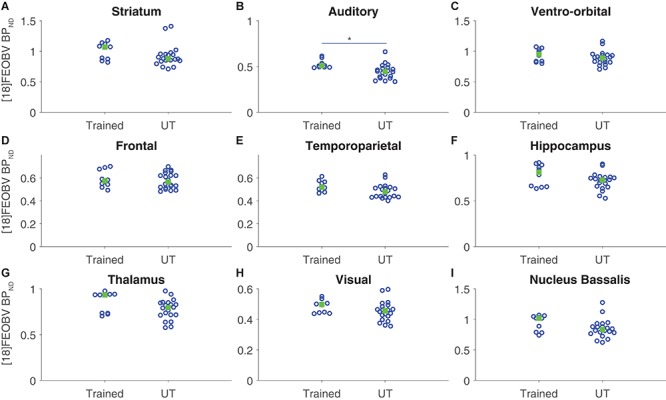
Region-specific cholinergic enhancement following auditory discrimination training assessed with [^18^F]FEOBV binding potential. Vertical scatter plots of mean regional [^18^F]FEOBV distribution per hemisphere for each ROI, organized by group. For each subject, BP_ND_ values for individual ROIs were calculated using the cerebellum as the reference region. Median values are marked with a green box. Multiple comparisons with group as predictor factor and ROI and hemisphere as within-subject (repeated) factors revealed a significant difference in [^18^F]FEOBV BP_ND_ in auditory cortex (*p* = 0.038) only (rest, all *p* ≥ 0.17, with Tukey–Kramer correction). **(A)** striatum, **(B)** auditory cortex, **(C)** ventro-orbital cortex, **(D)** frontal cortex, **(E)** temporoparietal cortex, **(F)** hippocampus, **(G)** thalamus, **(H)** visual cortex, **(I)** nucleus basalis. *p < 0.05.

[^18^F]FEOBV binding potentials were quantified for each ROI and compared between groups. For group comparisons, the average BP_ND_ in each ROI in each hemisphere was considered as a data point (two data points per rat/scan per ROI). A repeated measures model was used, where the repeated measures corresponded to the 18 individual ROIs per rat/scan, group was the predictor variable, and the within-subjects variables were ROI and hemisphere (left or right). There were no significant differences in overall [^18^F]FEOBV BP_ND_ as a function of group or hemisphere (both *p* ≥ 0.25, simple main effects test). Further group × ROI analysis revealed a significant increase in [^18^F]FEOBV BP_ND_ exclusively in the auditory cortex of the trained group, relative to controls (*p* = 0.038; all other ROIs, *p* ≥ 0.17, with Tukey–Kramer test for multiple comparisons; Figure 3).

**FIGURE 4:**
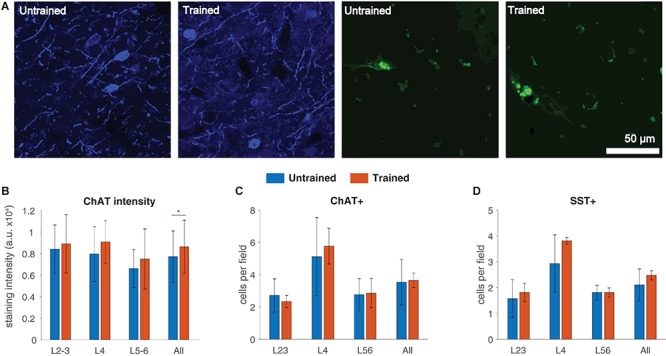
Increased intensity of ChAT staining in A1 following auditory discrimination training. **(A)** High-power microphotographs of A1 sections stained for choline acetyl-transferase (ChAT, *left*) and somatostatin (SST, *right*). **(B)** Increased overall ChAT staining intensity in the trained group (effects of group: *F*(1,21) = 4.72, *p* = 0.041; layer: *F*(2,21) = 3.44, *p* = 0.051; interaction, *F*(2,21) = 0.31, *p* = 0.73; two-way ANOVA). Group, number of subjects, high-power fields (hpf), and individual data points: Untrained, 6, 126, 446; Trained, 3, 63, 230. **(C)** ChAT+ cell count: two-way rmANOVA with group and layer as factors. Effects of layer: *F*(2,14) = 17.58, *p* = 0.0001; group: *F*(1,7) = 0.01, *p* = 0.9; interaction: *F*(2,14) = 0.44, *p* = 0.64. **(D)** SST+ cell count: two-way rmANOVA with group and layer as factors. Effects of layer: *F*(2,14) = 22.03, *p* < 0.001; group: *F*(1,7) = 0.81, *p* = 0.39; interaction: *F*(2,14) = 1.3, *p* = 0.3. Group, number of subjects, hpf for SST/ChAT+ cell counts: Untrained, 6, 126; Trained, 3, 63. Values shown are mean ± standard deviation. *p < 0.05.

To obtain histological confirmation of the effect of training on the cholinergic system, we compared the density of choline acetyl-transferase (ChAT) staining in A1 sections obtained from both untrained (*n* = 6) and trained rats (*n* = 3; Figure 4A, *left*). As reported earlier (Voss et al., 2016), ChAT density was significantly higher in trained rats (863.95 ± 245.87 optical density arbitrary units, a.u.) compared to untrained ones (772.67 ± 237.91 a.u., *p* = 0.041, two-way ANOVA; Figure 4B), consistent with both our above results and previous imaging findings (Parent et al., 2013b).

To determine whether increased density of ChAT staining might be related to increased cholinergic innervation onto A1, we performed quantitative analysis of the density of ChAT-positive (ChAT+) cells across layers. However, we did not find any significant differences in the average number of ChAT immunoreactive cells per A1 high power field (hpf) between trained and untrained rats (Figure 4C). Somatostatin-positive (SST+) cells, the second largest interneuron subpopulation, are the primary targets of cholinergic afferents to the cortex, and contribute to the neuromodulation of sensory processing during both passive sound exposure (Chen et al., 2015) and operant training (Fu et al., 2015). For this reason, we also quantified the density of SST+ cells per hpf (Figure 4A, *right*). In line with our results on ChAT+ cells, the control and experimental groups did not differ in overall nor layer-specific SST+ cell density (Figure 4D). Taken together, these results suggest that training-related changes in [^18^F]FEOBV binding detected by PET imaging were associated with an overall increase in cholinergic neurotransmission, without any layer-specific changes.

## Discussion

The purpose of the present study was to investigate how the aging rodent cholinergic system responds to auditory perceptual learning using a novel PET radioligand that selectively binds to VAChT. We showed that auditory perceptual learning increases cholinergic neurotransmission within the aged auditory cortex as assessed via both [^18^F]FEOBV PET imaging and immunochemistry. Our findings suggest that auditory perceptual learning significantly increased cholinergic neurotransmission in auditory cortex only, and not in any of the other ROIs. To our knowledge, this is the first use of [^18^F]FEOBV in rodents to demonstrate how perceptual learning can modulate the expression of ACh in a region-specific manner.

This increase in ACh binding following training sharply contrasts with the more typical age-related decline in binding observed with aging. Indeed, aging is associated with the degeneration of cholinergic cells (Perry, 1980; McGeer et al., 1984) and their projections (Gibson et al., 1981; Altavista et al., 1990). These age-related changes in cholinergic neurotransmission likely contribute to the attentional and cognitive deficits observed during aging (Everitt and Robbins, 1997; Schliebs and Arendt, 2011). This hypothesis is supported by converging evidence from various lines of research on the long-term effects of manipulating cholinergic neurotransmission in learning and attention. First, the activation of the cholinergic system during perceptual training leads to a long-lasting shaping of cortical circuits that forms the basis of learning (Rokem and Silver, 2013). Indeed, neurochemically boosting cholinergic transmission (Greuel et al., 1988; Furey et al., 2008; Rokem and Silver, 2010) and stimulating the basal forebrain (Greuel et al., 1988; Furey et al., 2008; Rokem and Silver, 2010) have both been shown to have a significant effect on both learning and the cortical processing of stimuli. Second, perceptual learning can itself produce long-lasting changes in perceptual abilities (Goldstone, 1998; Ohl and Scheich, 2005). Third, selective lesions of cholinergic neurons in the rat pedunculopontine tegmental nucleus resulted in attentional deficits that were proportional to the extent of neuronal loss (Cyr et al., 2015). Consequently, in general, ACh is believed to mediate voluntary attention, a major requisite for adult learning (Hasselmo and Bower, 1993; Sarter et al., 2005).

Recent studies have shown that, on shorter time-scales, fast central cholinergic responses observed in sensory cortex during learning might in fact convey reinforcement- and reward-elicited signals that enable the brain to associate prior events with behavioral outcome, thereby promoting local cortical plasticity and learning (Hangya et al., 2015; Liu et al., 2015). Of note, such precisely timed cholinergic signaling onto sensory cortex appears to be essential for sustained performance even after the completion of training (Kuchibhotla et al., 2017). Given the time scale of the present study, both types of cholinergic influence – on attention and reinforcement – are likely to have played a role in promoting plasticity and learning. However, future studies are warranted in order to disentangle the relative attentional and reinforcement effects of cholinergic input during perceptual learning.

As highlighted above, we found elevated cholinergic binding in the auditory cortex following perceptual learning. Although peak [^18^F]FEOBV levels are observed within the first 5 min post-injection in all brain regions, previous experiments have shown that relevant mapping of VAChT concentrations can be reliably obtained within a 60 min acquisition (Mulholland et al., 1998; Kilbourn et al., 2009). Furthermore, [^18^F]FEOBV binding distribution in the brain corresponds to the known anatomical distribution of cholinergic terminals (Kilbourn et al., 2009; Parent et al., 2012, 2013b). Moreover, [^18^F]FEOBV PET can detect cholinergic depletion, whether it be following lesions of the nucleus basalis (Cyr et al., 2014) or in association with aging (Parent et al., 2012), deficits that were also confirmed with ChAT immunostaining (Parent et al., 2013b). Consequently, current evidence suggests that [^18^F]FEOBV can reliably be used to assess both the integrity and the learning-induced changes of the ACh system.

Our anatomical findings of an overall ChAT staining intensity increase in the trained animals are consistent with our [^18^F]FEOBV PET findings, and suggest that training-induced plasticity in the cholinergic system was indeed characterized by an overall increase in A1 ACh neurotransmission. We did not find any layer-specific group differences in ChAT staining intensity, albeit a non-statistically significant increase was observed for all layers (see Figure 4). Although the present findings do not allow us to conclude on the matter, recent evidence suggests that the cholinergic control of cortical synaptic plasticity may be layer-specific (Bloem et al., 2014), whereby cholinergic stimulation produces opposite plasticity modulation effects on the superficial and deep cortical layers (Verhoog et al., 2016; Obermayer et al., 2017).

One of the more robust explanations of the role of ACh in perceptual learning posits that learning-related plasticity is achieved by a cholinergic-mediated shift in the cortical excitatory/inhibitory balance such that the input of afferent excitatory projections is favored over that from lateral intracortical connections (Hasselmo and Bower, 1993). A wealth of studies support this theory, and have specifically shown that cortical interneuron activity is modulated by cholinergic afferents (Kawaguchi, 1997; Fanselow et al., 2008; Kuchibhotla et al., 2017). In contrast to our ChAT staining intensity findings and our previous report of increased SST+ cell counts with auditory training (Voss et al., 2016), we found no significant changes in SST+ cell density in the present study. At least three factors might have contributed to this discrepancy. The first two factors relate to the strain and age of experimental subjects (Brown Norway rats, all ≤30 months in our previous study vs. LOU rats, all ≥30 months in the present investigation). Finally, although similar learning-related increases in SST+ cell density have been reported in young adult barrel cortex (Cybulska-Klosowicz et al., 2013), it is not clear whether perceptual learning is associated with neurogenesis in adult neocortex (Greene et al., 2019). The latter observation would also explain, at least in part, the unchanged ChAT+ cell counts in our trained group. In light of these mixed results, further studies are needed to clarify whether there is a causal link between cholinergic neurotransmission and SST+ cell density. Future studies may also explore the possible differential recruitment of cortical interneurons in a layer-specific manner by cholinergic inputs and their contribution to auditory perceptual learning.

Even though the present study is the first use of [^18^F]FEOBV in rodents to demonstrate how perceptual learning can modulate the expression of ACh, some limitations are worthy of mention. The primary limitation is the cross-sectional design. The rationale behind this design choice stems from the fact that this study was dependent on leftover [^18^F]FEOBV synthesized for ongoing human studies, making it difficult to predict when the next scans would occur. And although we were still able to identify perceptual learning-related increases in VAChT expression, a longitudinal design would yield a more accurate picture of ACh system changes as a function of learning. Furthermore, a longitudinal design would allow for a direct correlation between ACh binding and learning efficiency. Future studies may also want to compare [^18^F]FEOBV binding prior to and immediately after training to investigate the short-term changes produced in the cholinergic system by perceptual learning.

## Conclusion

In conclusion, we believe our study provides proof of principle data that PET combined with the [^18^F]FEOBV radioligand can be used to assess changes in cholinergic neurotransmission induced by neuroplastic learning processes.

## Data Availability Statement

The datasets generated for this study are available on request to the corresponding author. Data have been deposited in the online open access repository Mendeley Data: https://data.mendeley.com/datasets/ddm47dxms7/1.

## Ethics Statement

The animal study was reviewed and approved by the Montreal Neurological Institute Animal Care Committee.

## Author Contributions

JC-F and ÉD-V-S: conceptualization. MK, KR, DR, and PR-N: methodology. JC-F, MK, and MT: formal analysis. JC-F, MK, MT, JC, and KR: investigation. PG, DR, PR-N, and ÉD-V-S: resources. PV: writing – original draft. JC-F, PV, MT, and ÉD-V-S: writing – review and editing. PR-N and ÉD-V-S: supervision. ÉD-V-S: funding acquisition.

## Conflict of Interest

The authors declare that the research was conducted in the absence of any commercial or financial relationships that could be construed as a potential conflict of interest.

## Footnotes

1 http://www.bic.mni.mcgill.ca/ServicesSoftware/MINC

2 http://rsb.info.nih.gov/ij/
